# A Small Molecule p75^NTR^ Ligand, LM11A-31, Reverses Cholinergic Neurite Dystrophy in Alzheimer's Disease Mouse Models with Mid- to Late-Stage Disease Progression

**DOI:** 10.1371/journal.pone.0102136

**Published:** 2014-08-25

**Authors:** Danielle A. Simmons, Juliet K. Knowles, Nadia P. Belichenko, Gargi Banerjee, Carly Finkle, Stephen M. Massa, Frank M. Longo

**Affiliations:** 1 Department of Neurology and Neurological Sciences, Stanford University, Stanford, California, United States of America; 2 Department of Neurology, University of North Carolina at Chapel Hill, Chapel Hill, North Carolina, United States of America; 3 Department of Neurology and Laboratory for Computational Neurochemistry and Drug Discovery, San Francisco Veterans Affairs Medical Center, and Department of Neurology, University of California San Francisco, San Francisco, California, United States of America; University of Queensland, Australia

## Abstract

Degeneration of basal forebrain cholinergic neurons contributes significantly to the cognitive deficits associated with Alzheimer's disease (AD) and has been attributed to aberrant signaling through the neurotrophin receptor p75 (p75^NTR^). Thus, modulating p75^NTR^ signaling is considered a promising therapeutic strategy for AD. Accordingly, our laboratory has developed small molecule p75^NTR^ ligands that increase survival signaling and inhibit amyloid-β-induced degenerative signaling in *in vitro* studies. Previous work found that a lead p75^NTR^ ligand, LM11A-31, prevents degeneration of cholinergic neurites when given to an AD mouse model in the early stages of disease pathology. To extend its potential clinical applications, we sought to determine whether LM11A-31 could reverse cholinergic neurite atrophy when treatment begins in AD mouse models having mid- to late stages of pathology. Reversing pathology may have particular clinical relevance as most AD studies involve patients that are at an advanced pathological stage. In this study, LM11A-31 (50 or 75 mg/kg) was administered orally to two AD mouse models, Thy-1 hAPP^Lond/Swe^ (APP^L/S^) and Tg2576, at age ranges during which marked AD-like pathology manifests. In mid-stage male APP^L/S^ mice, LM11A-31 administered for 3 months starting at 6–8 months of age prevented and/or reversed atrophy of basal forebrain cholinergic neurites and cortical dystrophic neurites. Importantly, a 1 month LM11A-31 treatment given to male APP^L/S^ mice (12–13 months old) with late-stage pathology reversed the degeneration of cholinergic neurites in basal forebrain, ameliorated cortical dystrophic neurites, and normalized increased basal forebrain levels of p75^NTR^. Similar results were seen in female Tg2576 mice. These findings suggest that LM11A-31 can reduce and/or reverse fundamental AD pathologies in late-stage AD mice. Thus, targeting p75^NTR^ is a promising approach to reducing AD-related degenerative processes that have progressed beyond early stages.

## Introduction

Degeneration of basal forebrain cholinergic neurons (BFCN) and their neurites is a major contributing factor to the cognitive dysfunction associated with Alzheimer's disease (AD). It precedes neuron loss and is associated with mild cognitive impairment, a predecessor of AD [1]. BFCN atrophy involves aberrant signaling through the neurotrophin receptor p75 (p75^NTR^). p75^NTR^ is abundantly expressed by these neurons in adulthood [2] and its signaling can positively or negatively affect the condition of BFCNs depending on the presence of several factors including co-receptors and ligands [3], [4]. For example, nerve growth factor (NGF) signaling through p75^NTR^ may promote cell survival or death depending on the presence of its high-affinity receptor TrkA [3], [4]. Neuronal degeneration also occurs when amyloid-β (Aβ)^1–40^ binds to p75^NTR^ [5], [6]. Moreover, BFCNs were found to degenerate when Aβ oligomers were delivered to the brains of wild-type (WT) but not p75^NTR^ deficient mice [7], and this degeneration was prevented by functionally removing the neurotrophin-binding domain of the receptor in an AD mouse model [8]. Together, these observations indicate that p75^NTR^ signaling plays a necessary role in enabling Aβ-induced degeneration and implicate it as an AD therapeutic target [9], [10], [11].

Accordingly, our laboratories have developed small molecule, non-peptide ligands of p75^NTR^ that promote survival-related signaling, inhibit Aβ-induced degenerative signaling, and reduce degeneration in Aβ-exposed neuronal cultures [10], [12], [13]. One such ligand, LM11A-31, is a water soluble amino acid derivative that reaches the CNS [14], [15] and has structural and chemical features similar to the NGF loop 1 domain, which is known to interact with p75^NTR^ [12]. LM11A-31 prevents BFCN atrophy and cognitive deficits in an AD mouse model when administration starts at early pathological stages of the disease, shortly after Aβ plaques appear [14], [16]. A frequent occurrence in AD drug development is that compounds eliciting promising results in preclinical models fail to produce positive outcomes in clinical trials. One potential reason for this failing is that, in most preclinical studies, therapy is initiated in animals exhibiting little or no pathology or behavioral symptoms, while clinical studies of AD therapies involve symptomatic patients at more advanced pathological stages [17], [18]. Until it is possible to reliably identify early stages of AD pathology in the clinical setting, a key strategy for preclinical testing of potential therapeutics will be to apply them to symptomatic animals with moderate to severe neuropathology which better corresponds to the clinical population. Therefore, this study examined whether LM11A-31 could arrest or reverse the degeneration of cholinergic neurites in two well characterized AD models, Thy-1 hAPP^Lond/Swe^ (APP^L/S^) and Tg2576 mice, with treatment beginning in mid- to late stages of disease progression characterized by the presence of abundant amyloid deposits, well established BFCN degeneration, and memory deficits apparent in multiple testing paradigms [14], [16], [19], [20], [21].

## Materials and Methods

### Ethics statement

All animal procedures were conducted in accordance with the National Institutes of Health *Guide for the Care and Use of Laboratory Animals*. Studies were performed at Stanford University with protocols approved by its Institutional Animal Care and Use Committee and at the Palo Alto Veteran's Administration Hospital with approval of its Committee on Animal Research. Protocols included efforts to minimize animal suffering and numbers of mice used.

### AD mouse models

Two mouse lines were used in these studies: 1) Thy-1 hAPP transgenic line 41 C57BL/6 which over-express human amyloid precursor protein (APP) 751 (hAPP751) containing the London (V717I) and Swedish (K670M/N671L) mutations under the murine Thy1 promoter [19], which will herein be referred to as APP^L/S^; and 2) Tg2576, which over-express the 695 amino acid human isoform of APP with the “Swedish” mutation [22].

### p75^NTR^ ligand

LM11A-31 [2-amino-3-methyl-pentanoic acid (2-morpholin-4-yl-ethyl)-amide] is a water soluble isoleucine derivative (MW 243.3); for structure and detailed pharmacological characterization see Massa et al. (2006) and Knowles et al. (2013). LM11A-31 was custom manufactured by Ricerca Biosciences (Painesville, OH) at >97% purity, as assessed by liquid chromatography/mass spectroscopy (LC-MS/MS) analysis.

### LM11A-31 treatment

**Figure 1:**
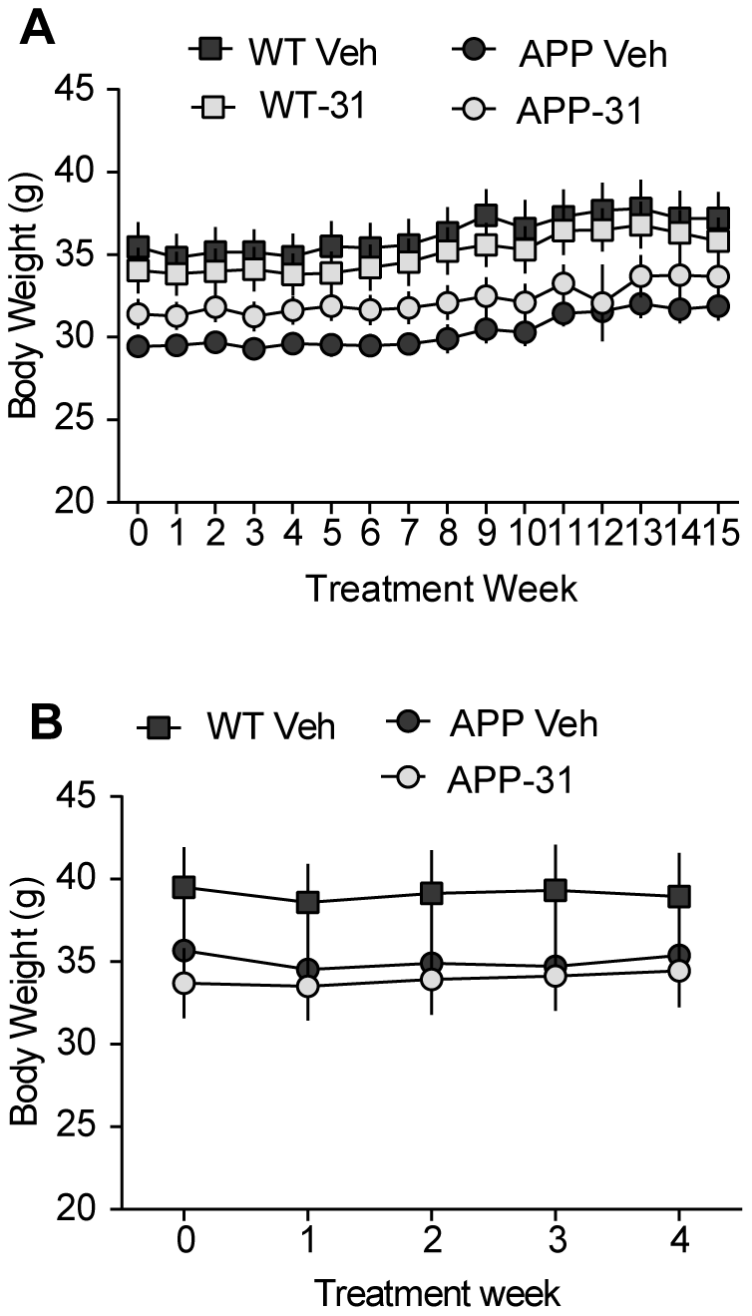
Body weight is not affected by treatment with LM11A-31 in male APP^L/S^ mice. Body weights of (***A***) 9–11 month old male APP^L/S^ mice treated with LM11A-31 (50 mg/kg) for 3 months (WT Veh, n = 9 mice; WT-31, n = 10; APP Veh, n = 10; APP-31, n = 9) and (***B***) 13–14 month old male APP^L/S^ mice treated with LM11A-31 (50 mg/kg) for 1 month (WT Veh, n = 5 mice; APP Veh, n = 4; APP-31, n = 5). APP mice weighed significantly less than WT mice in both of the age groups examined. LM11A-31 did not affect body weight at either age or genotype. Statistical significance was determined using repeated measures ANOVA with Dunnett's post-hoc test.

Both lines of transgenic mice and their gender- and age-matched non-transgenic (WT) littermates were randomly assigned to either vehicle or LM11A-31 treatment groups. For male APP^L/S^ mice, treatment started at two different ages: 1) 6–8 month old mice were treated for 3 months prior to euthanizing at 9–11 months old (“mid- stage pathology” cohort), and 2) 12–13 month old mice were treated for 1 month and euthanized at 13–14 months of age (“late-stage pathology” cohort). For both age groups, LM11A-31 was dissolved in sterile water and administered daily by oral gavage at 50 mg/kg/day. This dose was chosen based on positive effects of the ligand on histological and behavioral endpoints in previous studies from our laboratory [14], [16]. Furthermore, previous pharmacokinetic studies indicated that LM11A-31 (50 mg/kg) delivered daily by oral gavage for 2 weeks resulted in brain concentrations of 1904 nM [14], which greatly exceeds the *in vitro* effective dose [12]. Food was withheld 4 hours prior to dosing to aid in compound absorption. Vehicle-treated mice received an equivalent volume per weight of sterile water by the same route. Since LM11A-31 did not significantly affect any measure analyzed in the 6–8 month old WT mice, this group was not included in the study using 12–13 month old mice. Starting at the first week of treatment, APP^L/S^ mice weighed significantly less than WTs at both of the age groups studied, as neuropathology is present at the ages that treatment was started [14], [16], [19] (**Figure 1**). The body weights of the APP^L/S^ or WT mice in the LM11A-31groups did not differ from those of their respective genotypes given vehicle before or during treatment.

**Figure 2:**
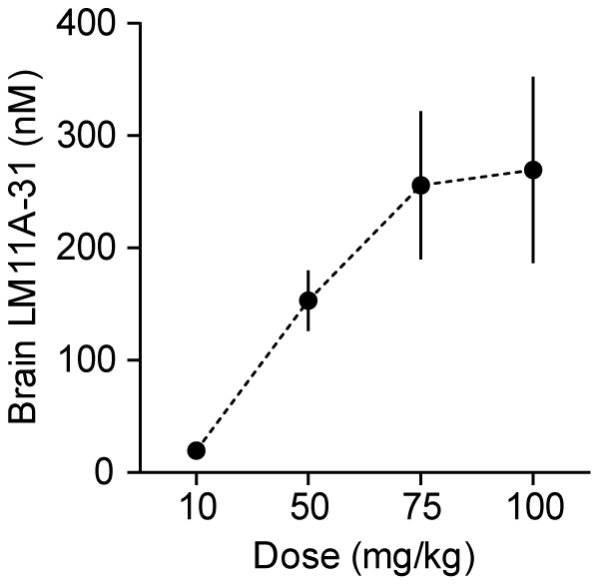
Brain concentrations of LM11A-31 delivered *ad libitum* in drinking water of C57BL/6 mice. LM11A-31 was given to C57BL/6 mice *ad libitum* in drinking water for 3 weeks at targeted doses of 10, 50, 75 and 100 mg/kg/day (n = 3–4 mice/dose). Brain concentrations were determined by LC-MS/MS by Absorption Systems (Exton, PA).

**Figure 3:**
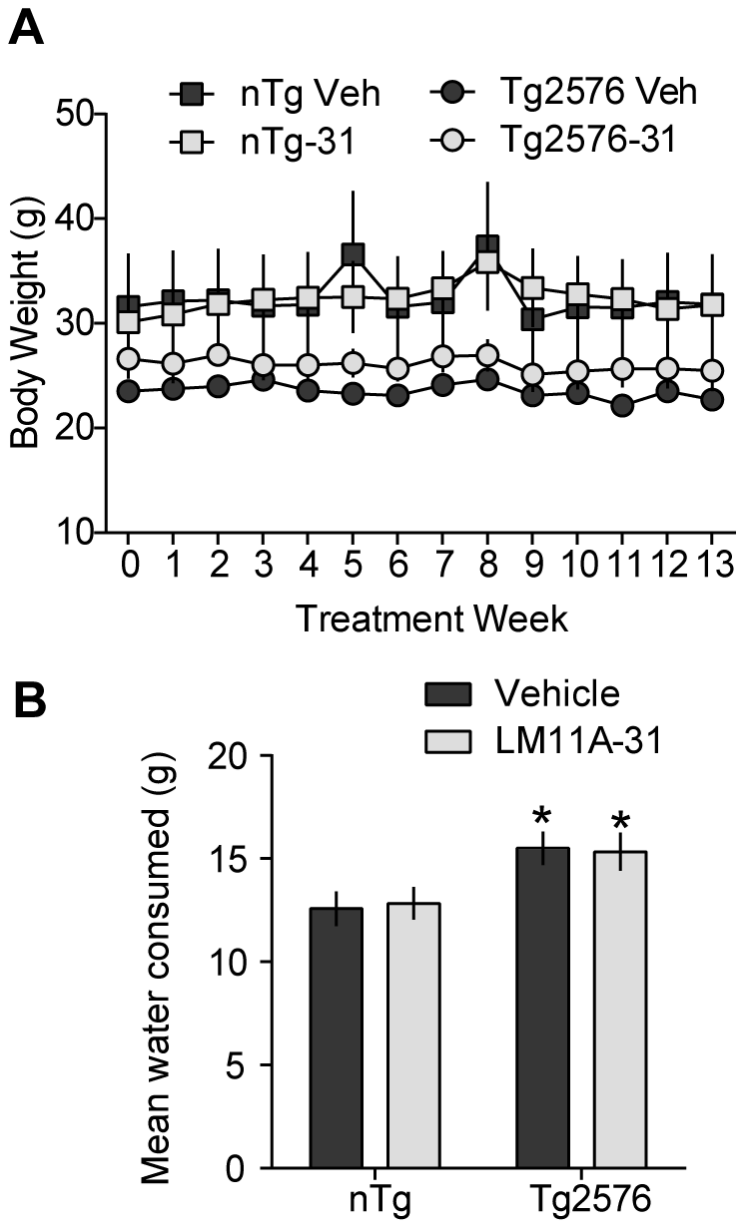
Body weight and water consumption are not affected by LM11A-31 treatment in Tg2576 mice. (***A***) Body weight of 17 month old female Tg2576 mice treated with LM11A-31 for 3 months (nTg-Veh, n = 7 mice; Tg2576-Veh, n = 6; Tg2576-31, n = 5; nTg–LM11A-31, n = 4). Starting at treatment week 6, Tg2576 mice weighed less than WT mice; LM11A-31 had no effect on this measure. Statistical significance was determined using repeated measures ANOVA with Dunnett's post-hoc test. (***B***) Average grams of water consumed by Tg2576 mice over 3 days was significantly greater than nTg mice; LM11A-31 had no effect on this measure. Statistical significance was determined using two-tailed Student's t-test. *p≤0.05 vs. nTg Veh.

For treatment of Tg2576 mice, LM11A-31 was delivered via drinking water. To determine dosing, LM11A-31 was administered to C57BL/6 mice *ad libitum* in drinking water for 3 months at targeted doses of 10, 50, 75 and 100 mg/kg/day (n = 3–4 mice/dose). Brain concentrations of the ligand were determined by LC-MS/MS at Absorption Systems (Exton, PA; for methods see [14]). A targeted dose of 75 mg/kg/day produced a brain concentration of 256±66 nM. This concentration was higher than that resulting from 10 and 50 mg/kg/day doses and the concentration (100 nM) that provided neuroprotection in *in vitro* studies [13], but very similar to that seen with 100 mg/kg/day (**Figure 2**). Therefore, 75 mg/kg/day was selected as the target dose for the Tg2576 study. Female Tg2576 mice and their non-transgenic (nTg) littermates were allowed *ad libitum* access to drinking water containing LM11A-31 (0.6 mg/ml; targeted dose 75 mg/kg) for 3 months starting at 14 months of age. Water bottles were refilled every 3 days and were weighed before and after filling to estimate doses received. Starting from treatment week 6, Tg2576 mice weighed significantly less than nTg mice; LM11A-31 did not significantly affect the body weights of either genotype (**Figure 3A**). Tg2576 mice consumed significantly more water than nTg mice (**Figure 3B**) and because of their lower body weights those in the treatment group received higher mean daily doses of LM11A-31. Actual mean dose per treatment group in mg/kg/day was: 79 for nTg and 119 for Tg2576 mice.

### Immunohistochemistry

After treatment, all mice were deeply anesthetized with avertin (2,2,2,tribromoehthanol; 425 mg/kg) and perfused with heparinized saline. Brains were removed, post-fixed in 4% paraformaldehyde for 24 hours at 4°C, and then cryoprotected in 30% sucrose. Brains were sectioned (40 µm, coronal) using a freezing microtome. Free-floating sections (every 8^th^ section in a 1 to 16^th^ series) were processed for immunohistochemical localization of choline acetyltransferase (ChAT; 1∶600; Millpore, Billerica, MA), as a marker for BFCNs, or p75^NTR^ using an antibody that recognizes the extracellular domain of the receptor (1∶800; Neuromics, Edina, MN). Sections were incubated in the Vectastain Elite ABC kit (Vector Laboratories, Burlingame, CA) solution and visualized with a diamino-benzidine substrate kit (Vector). Amyloid plaques were labeled with 1% Thioflavin-S (ThioS) stain.

### Microscopy and quantitative analysis

All immunostaining was visualized with a Leica DM 5000B or a Zeiss AxioImager M2 light microscope. Digitized images were acquired with the latter microscope using AxioVision software v4.7. Images for figures were acquired with a Zeiss AxioImager M2 light microscope using a 10, 20 or 63× oil objective and AxioVision software v4.7. All quantitative analyses were performed by experimenters that were blind to the treatment and genotype conditions.

In the basal forebrain, ChAT immunolabeling was viewed with a 40× objective and, using methods adapted from previous work [8], [14], [16], was evaluated in the vertical limb of the diagonal band of Broca (VDB), which was defined as the area below the anterior commissure (anterior part) at the rostro-caudal level of the islands of Calleja and before the emergence of the horizontal diagonal band (∼Bregma 0.98 mm [23]). After this anatomical criteria was applied only one section qualified for analysis when every 8^th^ section was processed. Brain sections were analyzed with Neurolucida v8 image analysis software (MBF Bioscience) using an unbiased automated scanning procedure (Meander Scan) that allows fields within a user-defined contour to be seen without overlap. In every third field, all neurons, and processes originating from them, were manually traced while focusing in the z-plane throughout the section to accurately assess volume and branching and to clearly discern the origin and end of neurites emanating from a particular cell. The software program moves the field of view to track the neurite throughout the VDB and realigns the tracing ensuring that the neurite is traced in its entirety in the x- and y-planes. Neurolucida Branched Structure Analysis was used to compute the volume, length, surface area, and branching order of the neurites.

Using methods previously established in our laboratory [14], ChAT immunostaining and ThioS staining in the motor and primary somatosensory cortices (between ∼Bregma 1.18–0.74 [23]) was visualized with a 10× objective and one digitized image was taken from each side of the brain in 3 sections per animal for a total of 6 fields/animal (sample field = 900×670 µm^2^). The same sample fields were analyzed for both stains. Clusters of ChAT-stained dystrophic neurites were manually traced by outlining their perimeter using Neurolucida v8 and the Branched Structure Analysis function was used to compute the area. ThioS staining was quantified from the digital images using histogram thresholding in Image Pro Plus v6.3 software (Media Cybernetics). The threshold was set manually to identify dense immunolabeling that was distinct from the background. The immunostained area was expressed as a percentage of the total area analyzed.

p75^NTR^-immunolabeling was visualized with a 20× objective in 2 sections per mouse in the basal forebrain (both medial septum and VDB), defined as the area above and below the anterior commissure (anterior part) at the rostro-caudal level of the islands of Calleja and before and just after the emergence of the horizontal diagonal band (∼Bregma 0.98–0.86 mm [23]). Three to four non-overlapping images were taken per section each using a 450×335 µm^2^ sample field. p75^NTR^-immunostained soma and neurites were automatically thresholded after background subtraction using ImageJ. The immunostaining area and density of both soma and neurites was measured and the number of soma was manually counted.

### Statistical analyses

GraphPad Prism v5 (GraphPad Software, San Diego California) was used to determine significance using a one-way Analysis of Variance (ANOVA) with Dunnett's multiple comparison post-hoc test and, when appropriate, a two-tailed Student's t-test. For planned comparisons in which a direction of difference was predicted, a one-tailed Student's t-test was used. A 2×2 contingency table with a Fisher's Exact test was used to determine the statistical significance of analyses with categorical variables (*e.g.* presence or absence of dendrite branching). For body weights, a repeated measures ANOVA with Dunnett's post-hoc test was used. Results are expressed as group mean ± SEM and statistical significance was set at p≤0.05.

## Results

### Effects of LM11A-31 on p75^NTR^ levels in basal forebrain of APP^L/S^ mice

**Figure 4:**
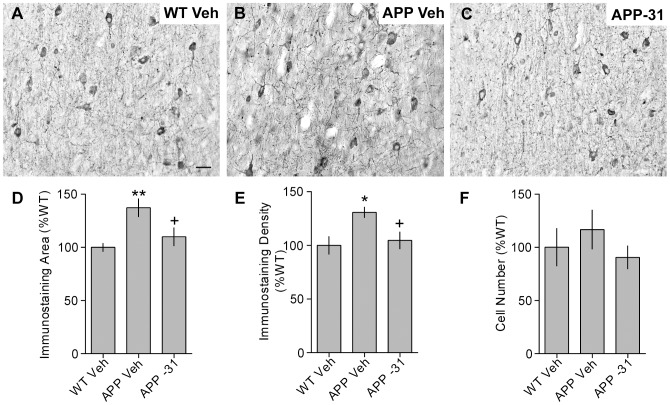
LM11A-31 normalizes increased p75^NTR^ levels in the basal forebrain of late-stage APP^L/S^ mice. Representative photomicrographs show p75^NTR^ immunostaining in the vertical limb of the diagonal band of the basal forebrain in (***A***) WT Vehicle (Veh), (***B***) APP^L/S^ (APP) Veh, and (***C***) APP LM11A-31 (-31) mice at 13–14 months old. Scale bar = 40 µm. Quantitative analysis showed that, at this age, the (***D***) area occupied by (^**^p≤0.01) and (***E***) the density (^*^p<0.05) of p75^NTR^ immunostaining is increased in APP Veh compared to WT Veh mice, while the increase in the number of p75^NTR^-stained cells was not statistically significant. LM11A-31 normalized p75^NTR^ levels (^+^p≤0.05 vs. APP Veh for both area and density). Statistical significance was determined using a one-way ANOVA with Dunnett's post-hoc test (WT Veh, n = 5 mice; APP Veh, n = 4; APP-31, n = 5).

Since levels of p75^NTR^ have been shown to increase in AD patients [24], [25] and mouse models [26], [27] with late-stage pathology and p75^NTR^ is the target of LM11A-31, we determined whether p75^NTR^ immunostaining was increased in the basal forebrain of APP^L/S^ mice at the ages involved in this study, and if levels were affected by the ligand, which could suggest target engagement. P75^NTR^ immunostaining did not differ between 9–11 month old APP^L/S^ and WT mice given vehicle and LM11A-31 treatment had no effect on this measure (data not shown). In contrast, in late-stage APP^L/S^ mice (13–14 months of age), the area occupied by p75^NTR^ staining associated with neurons and neurites was significantly increased by 37±8.5% and density was elevated by 31±5.2%, compared to WT mice (**Fig. 4A–E**). The number of p75^NTR^-stained cells increased slightly but this difference was not statistically significant (**Fig. 4F**). The increased area of p75^NTR^ immunostaining is most likely due to increased p75^NTR^ levels in neurons/neurites since the staining density significantly increased but the number of p75^NTR^-stained cells did not. Furthermore, previous reports showed that BFCN number, as assessed with ChAT immunostaining, was unaltered in APP^L/S^ mice compared to WTs [8], [14]. LM11A-31 treatment inhibited the increases in p75^NTR^ immunostaining in the basal forebrain of APP^L/S^ mice. This finding is consistent with our prior *in vitro* studies demonstrating that LM11A-31, like NGF, induces p75^NTR^ endocytosis and proteolytic processing and that the latter also occurs after administration to APP^L/S^ mice [16]. Thus, p75^NTR^ is present in abundance on the cells of interest in this study, showing feasibility of the ligand acting through its intended target, and LM11A-31 treatment decreases p75^NTR^ levels, which is consistent with target engagement.

### LM11A-31 prevents progression of and/or reverses cholinergic neuritic dystrophy in mid-stage APP^L/S^ mice

Since we previously found that treating 3–5 month old male and female APP^L/S^ mice with LM11A-31 for 3 months slowed the progression of cholinergic neurite dystrophy [14], [16], we sought to determine if LM11A-31 could prevent the progression of, and/or reverse, cholinergic neurite dystrophy with treatment beginning at a mid-pathological stage of the disease (6–8 months old). APP^L/S^ mice develop dense amyloid deposits in the frontal cortex and hippocampus at 3–4 and 5–7 months of age, respectively [19], and amyloid deposition progressively increases thereafter. BFCN loss does not occur in APP^L/S^ mice at 6–8 months, but atrophy of cholinergic neurites is evident [1], [8], [14], [16]. For example, the length of BFCN neurites in APP^L/S^ mice is ∼30% shorter than those in WT mice at that age [14], [16]. Moreover, hippocampal projection fibers of BFCNs are modulated by p75^NTR^ in WT mice [28] as are ChAT-containing neurites in APP^L/S^ mice [8]. Finally, mild cognitive deficits are first seen in APP^L/S^ mice at 3 months [21], are significantly worse at 6–8 months, and then progressively increase [14], [16], [20], [21].

**Figure 5:**
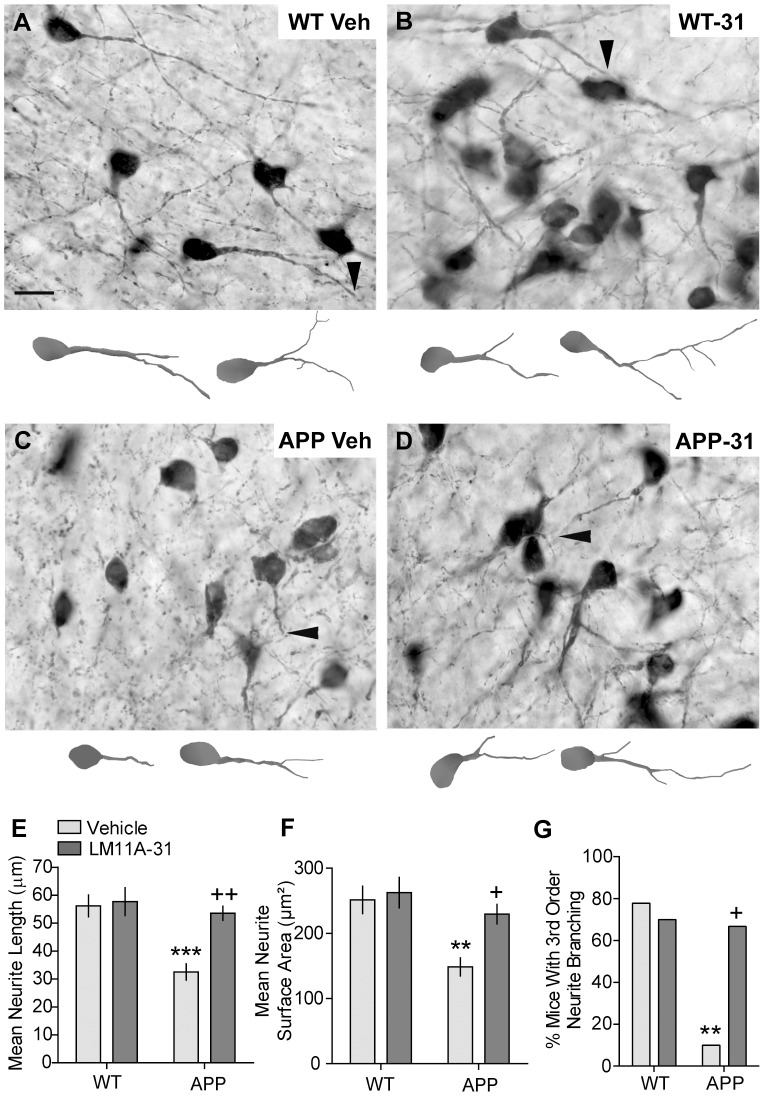
LM11A-31 prevents and/or reverses basal forebrain cholinergic neurite atrophy in mid-stage APP^L/S^ mice. Representative photomicrographs show ChAT-immunostained neurons in VDB of the basal forebrain of (***A***) WT Veh, (***B***) WT LM11A-31 (-31), (***C***) APP^L/S^ (APP) Veh, and (***D***) APP-31 mice at 9–11 months of age. Arrowheads indicate the distal part of neurites. Below each photomicrograph are reconstructed drawings from Neurolucida tracings of two ChAT-stained neurons per treatment group. The left drawing is the neurite and corresponding soma indicated by the arrowhead in the photomicrograph (orientations were altered). The right drawing is of a cell outside the field displayed in the photomicrograph but within the field analyzed. Scale bar in A = 20 µm and also applies to the line drawings. Quantification indicates that treating APP^L/S^ mice with LM11A-31 for 3 months increases the (***E***) length, (***F***) area occupied by, and (***G***) branching of BFCN neurites compared to those given vehicle. Statistical significance was determined using an ANOVA with Dunnett's post-hoc test and, for branching, a 2×2 contingency table with Fisher's exact test (WT Veh, n = 9 mice; WT-31, n = 10; APP Veh, n = 10; APP-31, n = 9). ^**^p≤0.01 and ^***^p<0.001 vs. WT Veh; ^+^p≤0.05 and ^++^p≤0.01 vs. APP Veh.

We examined the effects of LM11A-31 on the length and surface area of ChAT- immunolabeled neurites in the VDB because it provides a larger cholinergic projection to the cortex and hippocampus than the medial septum [29], [30]. The mean number of neurons analyzed per mouse was 71±3. The length of ChAT containing neurites of BFCNs appeared shorter in vehicle-treated mid-stage APP^L/S^ mice (9–11 months old) than any of the other groups (**Fig. 5A–D**). Quantitative analyses showed that ChAT-stained neurites were 39±5% shorter in vehicle-treated APP^L/S^ mice relative to their WT littermates (**Fig. 5E**) and their surface area was decreased by 41±6% (**Fig. 5F**). All mice of both genotypes had ChAT-positive neurites with 2^nd^ order branching, however, significantly fewer APP^L/S^ mice had neurites with ≥3^rd^ order branching compared to WT mice (**Fig. 5G**).

Treating mid-stage APP^L/S^ mice with LM11A-31 eliminated neurite degeneration as the length and surface area of their ChAT-stained neurites was equal to that of vehicle-treated WT mice (**Fig. 5B, C**). Furthermore, the reduction in branching complexity seen in vehicle-treated APP^L/S^ mice was markedly reduced with LM11A-31, as 6 of 9 treated APP^L/S^ mice had ChAT neurites with ≥3^rd^ order branching compared to 1 of 10 in the vehicle group (**Fig. 2G**). LM11A-31 did not affect the length, surface area or branching of BFCN neurites in WT mice (**Fig. 5D, E–F**).

**Figure 6:**
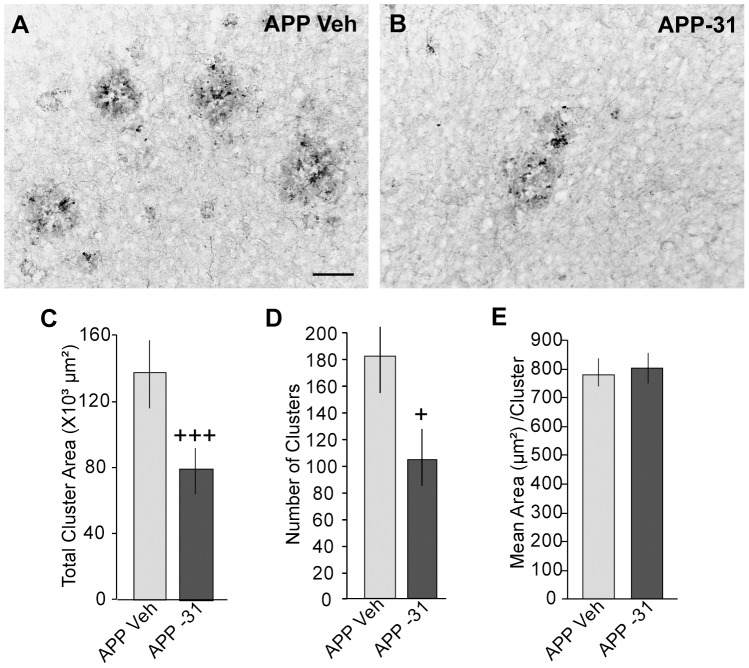
LM11A-31's effect on cholinergic dystrophic neurites in cortex of mid-stage APP^L/S^ mice. Representative photomicrographs show ChAT-immunostained dystrophic neurite clusters in the cortex of (***A***) APP^L/S^ (APP) Veh and (***B***) APP-31 mice. Scale bar in photomicrograph A = 50 µm. Quantitative analysis showed that LM11A-31 decreases the total area occupied by the clusters (***C***) by decreasing their number (***D***) but not the mean area per cluster (***E***). Statistical significance was determined using a two-tailed Student's t-test (APP Veh, n = 10; APP-31, n = 9). ^+^p = 0.03 and ^+++^p = 0.001 vs. APP Veh.

Since basal forebrain neurons are the main source of cholinergic innervation to the cortex [30] and cholinergic fibers become increasingly dystrophic with age in the vicinity of cortical amyloid plaques [14], [31], [32], [33], we examined the effects of LM11A-31 treatment on cholinergic dystrophic neurites in this region. Large clusters of dystrophic ChAT-stained neurites were prominent in the cortex of vehicle-treated APP^L/S^ mice (**Fig. 6A**), but were absent in WT mice (not shown). LM11A-31 decreased the total area occupied by ChAT-immunostained clusters in APP^L/S^ mice (**Fig. 6B,C**) by reducing the number of ChAT clusters (**Fig. 6D**) not the area per cluster (**Fig. 6E**).

LM11A-31 did not affect the percentage of the area occupied by ThioS staining in the cortex of APP^L/S^ mice (0.38±0.07% for vehicle group vs. 0.43±0.08% for LM11A-31). These results corroborate our previous studies indicating that fibrillar forms of Aβ were not affected by the ligand in APP^L/S^ mice at earlier disease stages [14], [16]. In addition, we previously reported that soluble Aβ(1-42) levels were not affected by the same LM11A-31 treatment [14]. Therefore, decreases in Aβ levels are unlikely to contribute to LM11A-31's prevention and/or reversal of ChAT neurite dystrophy.

### LM11A-31 reverses cholinergic neuritic dystrophy in late-stage APP^L/S^ mice

Given the prominent cholinergic neurite degeneration already present in APP^L/S^ mice at 6–8 months of age [14], [16] when treatment in the present study was initiated, the above findings that LM11A-31 treatment largely normalized cholinergic neurites suggested that the ligand, to a significant extent, reversed rather than simply slowed cholinergic neurite dystrophy. In order to further evaluate the existence of a reversal effect, we determined whether similar results could be obtained with a shorter (1 month) LM11A-31 treatment beginning in late-stage APP^L/S^ mice (12–13 months old) with more advanced pathology.

**Figure 7:**
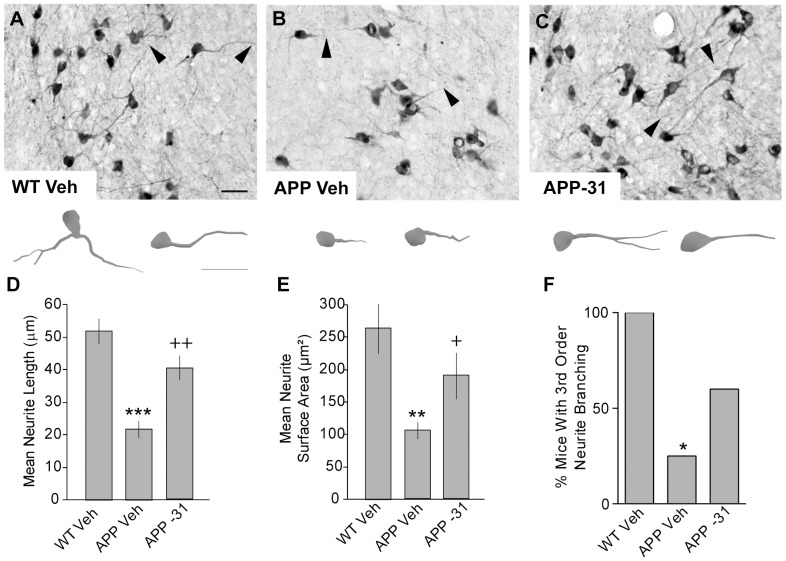
LM11A-31 prevents atrophy of cholinergic neurites in basal forebrain of late-stage APP^L/S^ mice. Representative photomicrographs show ChAT-immunostained neurons in the basal forebrain of (***A***) WT Veh, (***B***) APP^L/S^ (APP) Veh, and (***C***) APP-31 mice at 13–14 months of age. Arrowheads indicate the distal part of neurites. Scale bar in A = 40 µm. Below each photomicrograph are reconstructed drawings from Neurolucida tracings of two ChAT-stained neurons per treatment group indicated by the arrowhead in the photomicrograph. Scale bar in drawing under A = 40 µm. Quantitative analysis showed that treating late-stage APP^L/S^ mice with LM11A-31 for 1 month prevented the decreases in (***D***) length, (***E***) surface area, and (***F***) branching of cholinergic neurites in basal forebrain, although the latter measure did not reach statistical significance. Statistical significance was determined using an ANOVA with Dunnett's post-hoc test and/or two-tailed Student's t-test for BFCN neurite degeneration and, for branching, a 2×2 contingency table with Fisher's exact test (WT Veh, n = 5 mice; APP Veh, n = 4; APP-31, n = 5). ^*^p<0.05, ^**^p≤0.01 and ^***^p<0.001 vs. WT Veh; ^+^p<0.05 and ^++^p≤0.01 vs. APP Veh.

**Figure 8:**
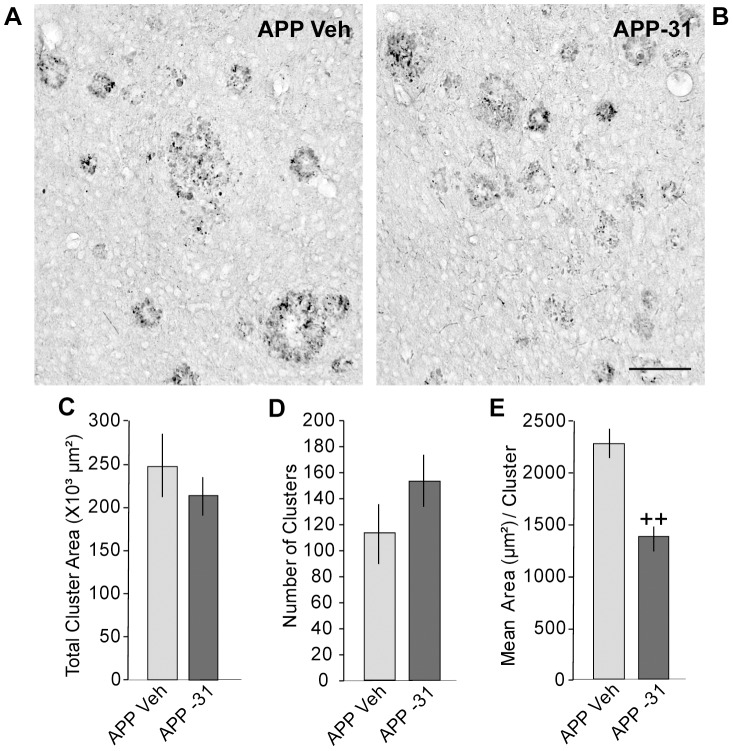
LM11A-31 prevents cholinergic dystrophic neurites in cortex of late-stage APP^L/S^ mice. Representative photomicrographs show clusters of cholinergic dystrophic neurites in the cortex of APP^L/S^ (APP) Veh (***A***) and APP-31 (***B***) mice. Scale bar in B = 50 µm. Quantitative analysis showed that LM11A-31 did not significantly affect the total area (***C***) or number (***D***) of clusters but did decrease the size or mean area per cluster (***E***). Statistical significance was determined using a two-tailed Student's t-test. ^++^p≤0.01 vs. APP Veh.

Within the 13–14 month old group, vehicle-treated APP^L/S^ mice had ChAT-stained neurites in the basal forebrain that were 58±5% shorter and occupied 60±4% less area than those of WT mice (**Fig. 7A–E**). Furthermore, all of the vehicle-treated WT mice at this age had neurites with ≥3^rd^ order branching, while only 1 of 4 APP^L/S^ mice had this characteristic (**Fig. 7F**). LM11A-31 prevented deficits in both neurite length and area in 13–14 month old APP^L/S^ mice compared to those given vehicle (**Fig. 7D,E**). It also increased the number of APP^L/S^ mice that had neurites with ≥3^rd^ order branching (3 out of 5 mice), however this increase did not reach statistical significance (**Fig. 7F**). In the cortex, LM11A-31 did not affect the total area occupied by or the number of ChAT neurite clusters but did significantly decrease the area per cluster (**Fig. 8A–E**). As in 9–11 month old APP^L/S^ mice, LM11A-31 did not affect the area of ThioS staining in the cortex of the late-stage APP^L/S^ mice (0.60±0.14% for vehicle group vs. 0.51±0.08% for LM11A-31). These results suggest that LM11A-31 may slow cluster expansion and coalescence and/or promote their disintegration.

**Figure 9:**
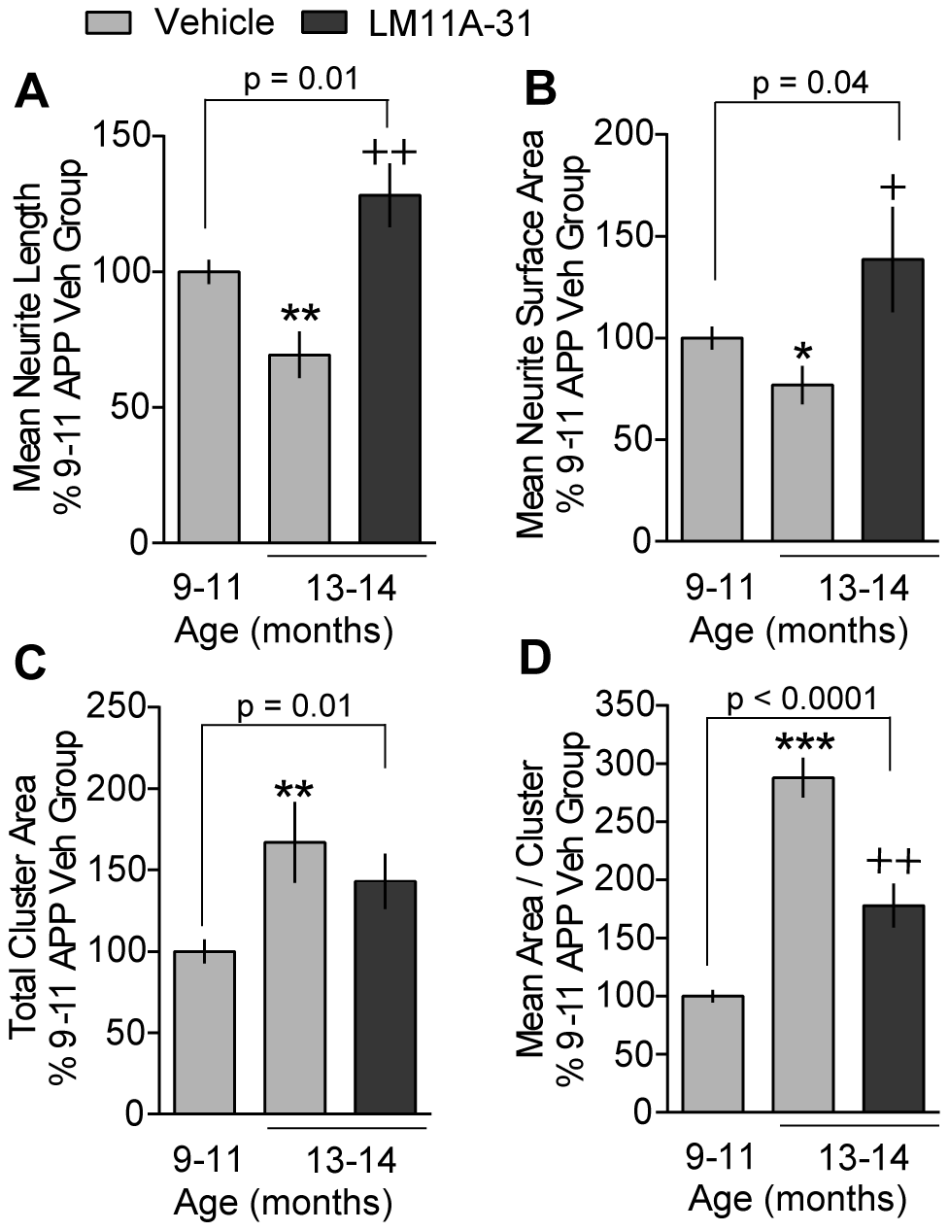
LM11A-31 reverses dystrophy of cholinergic neurites that occurs with aging in APP^L/S^ mice. Quantitative analysis of the effect of aging showed that 13–14 month old vehicle-treated APP^L/S^ (APP) mice had ChAT neurites in basal forebrain with decreased (***A***) length and (***B***) area and had dystrophic neurite clusters in cortex that occupied more area (***C***) and were larger (***D***) compared to the 9–11 month old APP Veh mice. In 13–14 month old APP mice, LM11A-31 increased the length and area of ChAT neurites in basal forebrain while decreasing the area of cortical dystrophic neurites compared to vehicle-treated mice (***A–D***; absolute numbers shown in Figs. 4 and 5). The effect of LM11A-31 in the basal forebrain was a reversal as neurite length (***A***) and area (***B***) were significantly greater in 13–14 month old APP-31 mice than 9–11 month old APP Veh mice. In the cortex, LM11A-31 did not reverse the area occupied by dystrophic neurites as 13–14 month old APP-31 mice still had significantly larger clusters than 9–11 month old APP Veh mice (***C, D***). Results are expressed as a percentage of the 9–11 month old APP Veh group. Statistical significance was determined using one-tailed Student's t-test (For 9–11 month old APP Veh n = 10; For 13–14 month group: APP Veh, n = 4; APP-31, n = 5). ^*^p≤0.05, ^**^p≤0.005 and ^***^p≤0.001 vs. 9–11 month old APP Veh; ^+^p≤0.05 and ^++^p≤0.01 vs. 13–14 month old APP Veh.

Aging significantly affected ChAT neurites in APP^L/S^ mice but not in WT mice. Vehicle-treated WT mice at 13–14 months of age had ChAT neurites in the basal forebrain that were nearly identical in length and the area they occupied compared to those in 9–11 month old mice. In contrast, as predicted for a progressive degenerative disease, 13–14 month old APP^L/S^ mice had ChAT neurites with reduced length and area compared to those in 9–11 month old APP^L/S^ mice (**Fig. 9A,B**). Moreover, the total area and size of ChAT-stained dystrophic neurite clusters in the cortex were increased substantially in the 13–14 versus 9–11 month old APP^L/S^ mice (**Fig. 9C,D**). However, the number of cortical dystrophic neurite clusters and ChAT neurite branching did not significantly change between these age groups (compare **Figs. 6** and **8**, **5** and **7**).

Notably, the 13–14 month old APP^L/S^ mice treated with LM11A-31 had ChAT neurites with significantly greater length, area (**Fig. 9A,B**), and branching (p = 0.02, one-tailed Chi-square) than 9–11 month old vehicle-treated APP^L/S^ mice. This finding suggests a reversal of pathology rather than a mere slowing of progression. The reduced area of cortical ChAT neurite clusters seen in the 13–14 month old APP^L/S^ mice given LM11A-31 does not necessarily include a reversal effect as the total area and area per cluster were still significantly higher than in the 9–11 month old vehicle-treated APP^L/S^ mice (**Fig. 9C,D**).

### Cholinergic neuritic dystrophy is ameliorated with LM11A-31 treatment in mid- to late-stage Tg2576 mice

To further assess the robustness of the effect of LM11A-31 in slowing progression of degeneration, we determined whether the ligand would be effective in reducing neurite degeneration in another well-characterized AD mouse model, Tg2576 mice. LM11A-31 was administered to female Tg2576 mice and their nTg littermates for 3 months starting at 14 months of age. In these mice, cognitive deficits are seen at 3 months of age and are progressive [34], [35], [36]. Insoluble Aβ levels incrementally increase beginning at 6 months of age [37] and Aβ deposition is evident at 11 months [22], [38]. Cholinergic dystrophic neurites are not seen at 8 months of age but occur in the cortex by 16 months [39].

**Figure 10:**
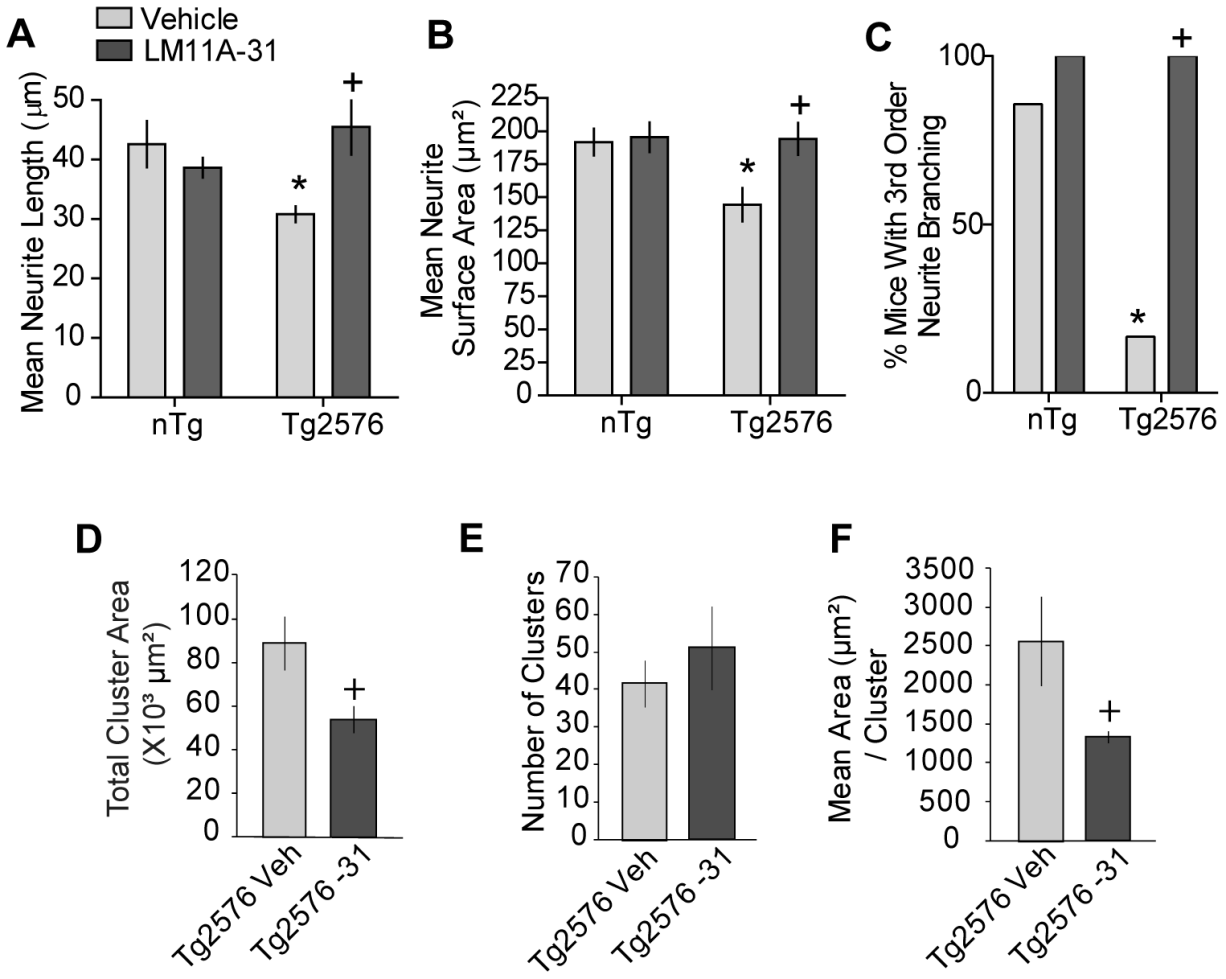
LM11A-31 prevents dystrophy of cholinergic neurites in mid- to late-stage female Tg2576 mice. Quantitative analysis showed that treating female Tg2576 mice starting at 14 months of age with LM11A-31 for 3 months prevented and/or reversed the decreases in (***A***) length, (***B***) surface area, and (***C***) branching of BFCN neurites. This treatment also decreased the total area (***D***) occupied by the clusters of cholinergic dystrophic neurites in the cortex and the mean area per cluster (***F***) but did not affect the number (***E***). Statistical significance was determined using an ANOVA with Dunnett's post-hoc test for BFCN neurite degeneration, a 2×2 contingency table with a Fisher's exact test for branching, and a two-tailed Student's t-test for clusters (nTg-Veh, n = 7 mice; Tg2576-Veh, n = 6; Tg2576-31, n = 5; nTg–LM11A-31, n = 4). ^*^p≤0.05 vs. nTg veh; ^+^p≤0.05 vs. Tg2576-Veh.

In comparison to nTg mice, vehicle-treated Tg2576 mice at 17 months old had shorter ChAT-stained neurites in the basal forebrain that occupied less area (**Fig. 10A,B**). These deficits were prevented in Tg2576 mice given LM11A-31, which had cholinergic neurites resembling those of nTg mice. Dendrite branching complexity was also decreased in vehicle-treated Tg2576 mice but not in those given LM11A-31, compared to nTg mice (**Fig. 10C**). Finally, LM11A-31 did not affect the number of cholinergic dystrophic neurite clusters in the cortex, but significantly reduced the total area they occupied as well as the mean area per cluster (**Fig. 10D–F**). LM11A-31 did not affect any measure in nTg mice. These findings confirmed the ability of LM11A-31 treatment to prevent neurite degeneration in a second mouse model. The extent to which treatment might reverse degeneration in this model will require studies at later disease stages.

## Discussion

This study showed that administering the p75^NTR^ ligand LM11A-31 to APP^L/S^ mice with mid- to late-stage pathology slowed the progression of cholinergic neurite degeneration in the basal forebrain and cortex and this effect was confirmed in the Tg2576 model. Importantly, LM11A-31 reversed degeneration when it was administered for a brief duration (1 month) and initiated at an age (12–13 months) that was about four times older than the age of detectable pathology onset (3–4 months old). To our knowledge, this report is the first to demonstrate that a CNS penetrating small molecule can function at a specific target to reduce and/or reverse fundamental AD pathologies in late-stage AD mice.

The length and branching complexity of ChAT-stained dendrites in the basal forebrain was reduced in mid- and late-stage APP^L/S^ mice as well as Tg2576 mice. LM11A-31 reversed these changes. Dendritic abnormalities and decreases in higher order branching have also been observed in the hippocampus, basal forebrain, and/or cortex of Tg2576 mice [40] and in other AD mouse models and patients [32], [41], [42], [43], [44]. Shorter and less branched dendrites are correlated with dementia [41], [45], most likely due to decreased neuronal connectivity, and may therefore contribute to the memory deficits described in APP^L/S^ and Tg2576 mice. LM11A-31 may have exerted its effects on dendritic length and complexity via activation of the PI3K/AKT pathway as the ligand increases AKT signaling [12] and this pathway has been shown to increase dendrite branching and alter dendritic arbor shape [46].

Dystrophy of cholinergic neurites in the basal forebrain and cortex is exacerbated in an age-dependent manner in APP^L/S^ mice. These results are consistent with those seen in other mouse models of AD [31], [32], [33]. Notably, LM11A-31 reversed the effect aging has on cholinergic neurite dystrophy and eliminated the age-related increase in p75^NTR^ levels in the basal forebrain of APP^L/S^ mice. This latter effect could be due to LM11A-31-induced receptor internalization and/or processing or it could be secondary to decreasing pathology, as numerous mechanisms of cellular injury, including those related to AD, up-regulate p75^NTR^ [3], [25], [47], [48]. The LM11A-31-induced decrease in p75^NTR^ levels may account, in part, for the reversal of cholinergic deficits by decreasing the abnormally high p75^NTR^/Trk ratio in the basal forebrain found in human AD and mouse models [25], [26], [47], [48]. The reversal effects in aged APP^L/S^ mice occur even when treatment is shortened to 1 month and initiated after appreciable pathology has developed. This reversal holds particular relevance to translating a small molecule p75^NTR^ ligand to the clinic, wherein AD patients, even at early clinical stages, would more likely be in pathologically advanced stages of disease prior to treatment.

LM11A-31-treated APP^L/S^ mice with mid-stage pathology had fewer dystrophic neurite clusters in the cortex that occupied a smaller area but did not decrease in size. Although a reversal effect did not occur in the cortex of late-stage APP^L/S^ mice, just 1 month of LM11A-31 treatment reduced cluster size without affecting their number or overall area. Perhaps a longer treatment time would have reversed the total area and size of the clusters to the levels of the 9–11 month old vehicle-treated APP^L/S^ mice. Nevertheless, reducing either the size or the number of dystrophic neurite clusters could be beneficial and an indicator of slowing degenerative processes.

The positive results achieved here with LM11A-31 further validate p75^NTR^ as a therapeutic target for AD. The ability to reverse neurite degeneration in the context of ongoing, late-stage Aβ accumulation is particularly notable in terms of the array of fundamental AD-related signaling mechanisms modulated by LM11A-31. p75^NTR^ and TrkA signaling stimulated by NGF binding are essential for BFCN function and survival, and this signaling is disrupted in AD brains [49], . Furthermore, degenerative signaling is augmented in AD due, in part, to increased p75^NTR^/Trk ratios [52], [53], [54], elevated proNGF levels [15], [55], [56], [57], and Aβ interactions either directly and/or indirectly with p75^NTR^ [58]. LM11A-31 competes with NGF and proNGF binding to p75^NTR^ but not TrkA [12], [15] and has a signaling profile that is distinct from NGF [12]. Our previous *in vitro* studies showed that LM11A-31 inhibited Aβ-induced degenerative signaling (including excess activation of GSK3β, cdk5 and JNK), activated survival signaling that is compromised in AD (AKT and NFκB), and prevented excess tau phosphorylation in a p75^NTR^-dependent manner [13]. These actions and the present *in vivo* results showing prevention and reversal of BFCN degeneration are consistent with the idea that LM11A-31 can bind to p75^NTR^ to simultaneously increase survival signaling while decreasing degenerative signaling to ameliorate mid- to late-stage AD-related pathology.

In all, these results suggest that targeting of p75^NTR^ is a promising approach to modulating AD-related degenerative mechanisms in a way that is sufficiently robust to affect pathological processes that have progressed beyond early stages. Future studies will include behavioral analyses to assess whether reversing these neuropathologies improves functional outcomes.
